# SARS-CoV-2 and Public Transport in Italy

**DOI:** 10.3390/ijerph182111415

**Published:** 2021-10-29

**Authors:** Giuseppina Caggiano, Francesca Apollonio, Francesco Triggiano, Giusy Diella, Pasquale Stefanizzi, Marco Lopuzzo, Marilena D’Ambrosio, Nicola Bartolomeo, Giovanna Barbuti, Giovanni Trifone Sorrenti, Pantaleo Magarelli, Domenico Pio Sorrenti, Vincenzo Marcotrigiano, Osvalda De Giglio, Maria Teresa Montagna

**Affiliations:** 1 Department of Biomedical Science and Human Oncology-Hygiene Section, University of Bari Aldo Moro, Piazza G. Cesare 11, 70124 Bari, Italy; giuseppina.caggiano@uniba.it (G.C.); francesca.apo@libero.it (F.A.); giusy.diella@uniba.it (G.D.); pasquale.stefanizzi@uniba.it (P.S.); marcolopuzzo@gmail.com (M.L.); marilena.dambrosio@uniba.it (M.D.); nicola.bartolomeo@uniba.it (N.B.); giovanna.barbuti@uniba.it (G.B.); osvalda.degiglio@uniba.it (O.D.G.); mariateresa.montagna@uniba.it (M.T.M.); 2 Local Health Unit BT, Department of Prevention, Food Hygiene and Nutrition Service, Barletta-Andria-Trani, 76125 Trani, Italy; giovanni.sorrenti@aslbat.it (G.T.S.); pantaleo.magarelli@aslbat.it (P.M.); d.sorrenti2@studenti.uniba.it (D.P.S.); vincenzo.marcotrigiano@aslbat.it (V.M.)

**Keywords:** SARS-CoV-2, COVID-19, public transport, surfaces

## Abstract

Although direct contact is considered the main mode of transmission of SARS-CoV-2, environmental factors play an important role. In this study, we evaluated the presence of SARS-CoV-2 on bus and train surfaces. From the buses, we took samples from the following areas: handrails used to enter or exit the bus, stop request buttons and handles next to the seats. From the trains, the sampled surfaces were handrails used to enter or exit the train, door open/close buttons, handles next to the seats, tables and toilet handles. SARS-CoV-2 was detected on 10.7% of the tested surfaces overall, 19.3% of bus surfaces and 2% of train surfaces (*p* < 0.0001). On the buses, the most contaminated surfaces were the handles near the seats (12.8%), followed by door open/close buttons (12.5%) and handrails (10.5%). Of the five analyzed transport companies, bus companies were the most contaminated, in particular, companies C (40%) and B (23.3%). A greater number of positive samples were found among those taken at 10:00 a.m. and 10:55 a.m. (45% and 40%, respectively). The presence of the virus on many bus surfaces highlights how the sanitation systems on public transport currently in use are not sufficient to limit the spread of SARS-CoV-2.

## 1. Introduction

On 11 March 2020, the WHO declared COVID-19, the disease caused by Severe Acute Respiratory Syndrome Coronavirus 2 (SARS-CoV-2), a pandemic. Since then, numerous researchers and scientific communities have studied the characteristics of the virus and approaches to control its spread [1,2]. The virus continues to circulate throughout the world. Globally, as of 27 September 2021, there have been 231,703,120 confirmed cases of COVID-19, including 4,746,620 deaths reported to the WHO. In Italy, 4,660,314 cases have been registered, along with 130,697 deaths SARS-CoV-2 [3]. Like other respiratory viruses, can be transmitted by breathing; airborne spread represents the primary diffusion route [4]. Usually, it is believed that respiratory viruses are directly transmitted via contact between an infected person and a susceptible person, or indirectly via contact with surfaces or objects contaminated by droplets or aerosols [4,5,6]. Droplets are particles with a diameter greater than approximately 100 µm, while aerosols are droplets with a diameter ranging from fractions of micrometers to 100 µm [6]. In particular, large, heavy droplets are deposited on surfaces, while aerosols float in the air long enough to be inhaled, even at a distance from the source (infected subject) [7,8]. Environmental factors and contaminated surfaces play an important role in the transmission of SARS-CoV-2, even if direct person-to-person contact is considered to be the main mode of transmission. The virus can remain active for a variable period on different surfaces, maintaining its infective potential [9]. Several environmental factors affect the virus’s survival on surfaces. These include temperature, humidity, salinity, pH, the medium or materials of the contaminated objects or surfaces, ventilation, airflow and ultraviolet radiation [10,11].

To date, numerous studies have evaluated the presence of SARS-CoV-2 in hospital settings [12,13,14], but there are very few studies evaluating the presence of the virus in the community [15]. To the best of our knowledge, the national and international literature relating to the research on the virus on various means of public transport is extremely limited [16,17]. Di Carlo et al. [16] evaluated the presence of SARS-CoV-2 in the air and on the surfaces within buses circulating in Abruzzo (Italy), while Moreno et al. [17] researched the virus RNA on public buses and Barcelona metro trains. The results of the first study never showed the presence of viral RNA on buses, while the second study showed the presence of RNA residues more commonly on the surface pads of the support bars than in the ambient air inside the vehicles, with higher concentrations of viral RNA fragments on buses rather than trains.

The aim of this study was to evaluate the presence of SARS-CoV-2 on the surfaces within public transport, and to determine which time of day corresponds to the highest levels of contamination.

## 2. Materials and Methods

The Decrees of the President of the Italian Republic, dated 3 November 2020 and 14 January 2021, have divided Italy into four zones—red, orange, yellow and white areas—based on the levels of risk of spreading the infection. The risk level of each region was based on parameters identified by the Italian National Institutes of Health: number of symptomatic cases, hospitalizations, new outbreaks, beds occupied in hospitals and deaths. 

In the red areas, with a high level of risk, it was forbidden to enter and leave the region, and to move within the territory, except for reasons of health, work needs and proven needs. Prohibition of circulation from 10 p.m. to 5 a.m., except for reasons of necessity, was implemented. In the orange zones, with a medium–high risk level, it was possible for the inhabitants of small municipalities to move among small cities for reasons of necessity. In the yellow areas, with a medium–low level of risk, circulation was allowed without limits on time, motivation or travel between regions. Finally, in the white areas, with a low level of risk, people were allowed to move in and out of the region, and all activities were reopened with the obligation to wear a mask in closed spaces and outdoors when it was not possible to maintain a distance.

In the period between 24 May 2021 and 3 June 2021, when the Apulia Region was in the yellow zone, a cross-sectional survey was performed, collecting a total of 300 surface sample swabs from buses and trains joining the main cities in the Apulia region (Bari, Foggia, Barletta, Trani and Andria). Swabs were taken on 10 buses and five train lines (two wagons per line) belonging to five different companies: 150 specimens from four bus companies (hereinafter referred to as A, B, C and D) and 150 from the main (unique) railway company (hereinafter referred to as E). For each means of transport, 15 swabs were collected from the surfaces considered most likely to come into contact with users. The following surfaces were sampled from the buses: handrails used to enter or exit the bus, stop request buttons and handles next to the seats. On the trains, the sampled points were handrails used to enter or exit the train, door open/close buttons, handles next to the seats, tables and toilet handles.

### 2.1. Environmental Sampling

Surface sampling was carried out using sterile swabs inserted into a plastic tube (Easy Surface Checking (ESC)–Neutralizing Rinse Solution (NRS); Liofilchem Srl, Roseto degli Abruzzi, Italy) containing 10 mL of transport medium. Flat and wide surfaces (e.g., tables) were buffered on a well-defined area (10 × 10 cm), using a delimiter, while small surfaces and curves (e.g., handles) were buffered on the available area. The swabs were then placed in their protective cases and transported to the laboratory in a special isothermal refrigerator at a controlled temperature (+4 °C) and immediately processed. The sampling was carried out during periods of high passenger transit (between 9:00 a.m. and 12:00 p.m.).

### 2.2. Molecular Analysis

The detection of the presence of SARS-CoV-2 was performed by Real-Time Reverse Transcription PCR (RT-PCR) [15,18]. Swabs were vortexed for 20 s and transferred under sterile conditions to a new 15 mL tube. Nucleic acids were extracted from 5 mL of NRS medium using the NucliSENS miniMAG semi-automatic extraction system with magnetic silica, according to the manufacturer’s instructions (bioMerieux, Marcy-l’Etoile, Lyon-France). The RNA was resuspended in 100 µL of elution buffer, and the extracts were kept at −20 °C. For the amplification of ORF-1ab gene (nsp14), a 25-µL mixture was prepared containing: 5 µL of RNA for each sample; 12.5 µL of 2 × Reaction Buffer supplied with AgPath-ID ™ One-Step RT-PCR Reagents (Applied Biosystems ™, Thermo Fisher, Waltham, MA, USA); 1 µL of 25× RT-PCR enzyme mix; 1 µL of forward primer (12.5 µM); 1 µL of reverse primer (22.5 µM); 1 mL of probe (6.25 µM); 1.83 µL of nuclease-free water (not DEPC-treated); and 1.67 µL Real-Time PCR Detection Enhancer (Applied Biosystems ™, Thermo Fisher, Waltham, MA, USA). The primer and probe sequences used were: CoV-2-F/ACA TGG CTT TGA GTT GAC ATC T; CoV-2-R/AGC AGT GGA AAA GCAT GTG G; and CoV-2-P/FAM-CAT AGA CAA CAG GTG CGC TC-MGBEQ [18]. RT-PCR experiments were conducted in duplicate using the CFX96 Touch Deep Well Real-Time PCR System (Applied Biosystem ™, Thermo Fisher, Waltham, MA, USA). Thermal cycling conditions were reverse transcription phase (50 °C for 30 min), inactivation of the RT phase (+95 °C for 10 min) and 45 amplification cycles (+95 °C for 15 s and +60 °C for 45 s). Cycle cut-offs for RT-PCR were used as indicators of the SARS-CoV-2 RNA copy number in samples. A cycle cutoff value of less than 40 was interpreted as positive for SARS-CoV-2 RNA.

### 2.3. Statistical Evaluation

All analyzed data were categorical, and summarized as count and percentages. The comparison of percentages between independent groups was performed using the Fisher exact test. A logistic regression was performed to evaluate the odds ratio of positive samples. The dependent variable was the positive results of SARS-CoV-2 RNA and the independent variables were: the type of transport (train or bus), the routes (divided into eleven classes relative to starting in town to arriving in town), the time of the run (treated as categorical, with 13 classes from 10:00 to 11:30), the surface of swab retrieval (handrails, handle, buttons and tables), the number of seats (classified as 25 or less vs. more than 25), the day of the week (classified as Monday, Tuesday or Thursday) and the company (anonymized). The model was run as univariable, that is, a single model for each independent variable. Then, using only variables that were statistically significant in the univariable analysis, a multivariable logistic model was run with a stepwise selection. A *p*-value lower than 0.05 was considered statistically significant. The analysis was performed by Medcalc.

## 3. Results

**Figure 1:**
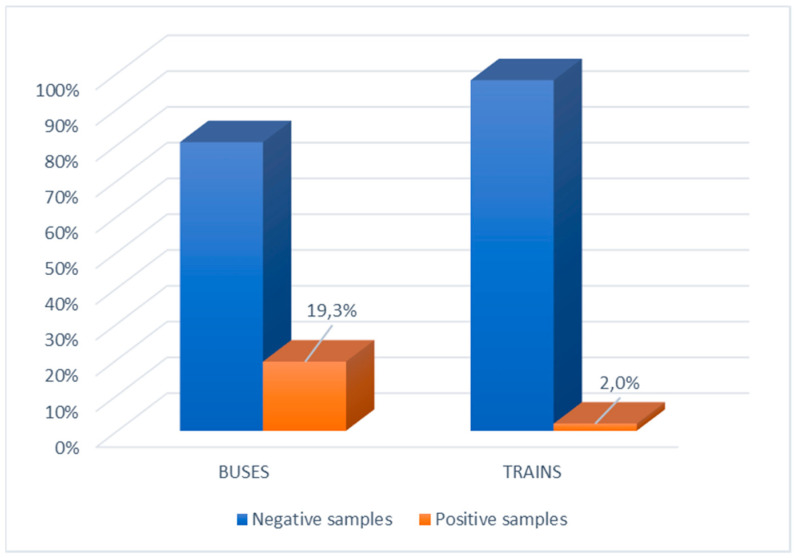
Distribution (%) of positive and negative samples taken from buses and trains.

The main characteristics of the survey are reported in Table 1. SARS-CoV-2 was detected in 10.7% (32/300) of the tested surfaces. On buses, 19.3% (29/150) of tested surfaces were contaminated, while 2% (3/150) of tested surfaces on trains were contaminated (*p* < 0.0001) (Figure 1).

Accordingly, the odds ratio in Table 2 for positive samples on buses was 11.73 (95% CI 3.5–39.5). On buses, the most contaminated surfaces were the handles near the seats (12.8%) followed by door open/close buttons (12.5%) and handrails (10.5%). The odds ratio of positive samples was not statistically significant (Table 2).

The percentage of virus-contaminated samples from bus handles and buttons was higher than that found on trains. The percentage of positive samples taken from door handles was 16% (12/75) on buses and 2% (1/49) on trains (*p* = 0.0149). The comparison of positive samples from seat handles was 30% (9/30) on buses and 2.1% (1/48) on trains (*p* = 0.00059). The difference between positive samples on buttons, 17.8% (8/45) on buses and 3.7% (1/27) on trains was not statistically significant.

With regard to the time slot most at risk, a greater positivity rate was found among the samples taken between 10:00 a.m. and 10:55 a.m. (45% and 40%, respectively), but the variable was not statistically significant either in the univariable or in the multivariable model (Table 2).

The results for Thursday showed a higher percentage of positive samples; moreover, this variable was statistically significant both in univariate analysis and in multivariable analysis. Data from Monday and Tuesday showed fewer positive results (Table 2). 

The occupied seats were observed as a statistically significant variable in the univariate analysis: the odds ratio suggested that the more crowded the transport, the lower the chance of positive samples. The results of the multivariable analysis did not show this variable as statistically significant.

The routes managed by the different companies showed a statistically significant positive effect: by comparing all routes, we observed that the routes involving the main town in BT county were most at risk of a positive result. The routes were removed based on the selection method for variables used in the multivariable model (Table 2).

**Figure 2:**
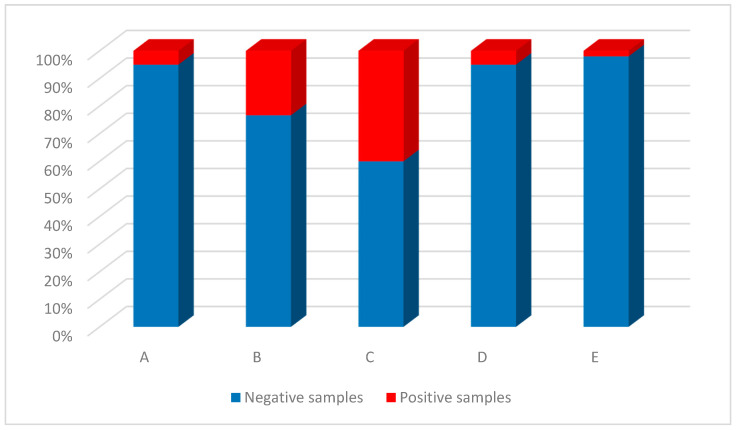
Distribution of positive and negative samples between five different transport companies.

Among the five transport companies analyzed (Figure 2), the four bus companies were more contaminated; specifically, company C had the greatest percentage of contaminated samples (40%), followed by company B (23.3%), A and D (5% for each). The train company (E) showed the presence of SARS-CoV-2 in 2% of the samples examined. The difference in positive samples from company C was statistically significant compared with companies A (*p* = 0.0075), D (*p* = 0.00049) and E (*p* < 0.0001). The difference in positive samples from company B was statistically significant with respect to companies A and D (both *p* = 0.024), and with respect to company E (*p* < 0.0001).

## 4. Discussion

To the best of our knowledge, this study is one of the first in Italy to evaluate the possible contamination of high-contact surfaces on public buses and trains. The research demonstrated the presence of SARS-CoV-2 in 10.7% of examined samples, mostly within buses. 

Although the virus can remain detectable for a variable period on different surfaces, its presence on a surface does not mean that the surface is a possible source of infection [19], unless the subject, after touching the contaminated surface, touches the mucous membranes of the respiratory tract with their hands.

Our study was conducted by applying only molecular investigations that highlight the genetic material of the virus, but not its viability. At the time of the investigation, we were not equipped to initiate cell cultures; however, we believe that finding genetic traces of SARS-CoV-2 is indicative of its circulation in the environment. Therefore, surfaces can be a source of infection if proper sanitation procedures are not applied.

The progressive evolution of the COVID-19 pandemic has changed the behavior of urban commuters to minimize their exposure to SARS-CoV-2. Some preventive measures (e.g., mandating the wearing of correctly fitted masks, social distancing during travel and adequate ventilation) can reduce the risk of transmission of the virus. To contain its spread, transport companies have developed new strategies, such as increasing the frequency of vehicle cleaning, increasing the use of hydroalcoholic solutions in cleaning and reducing the number of seats per row for greater social distancing. Previous studies have already highlighted how unclean hands can contribute to the contamination of inanimate surfaces [20]. 

It stands to reason that all of these approaches taken together (i.e., greater frequency of cleaning, a more effective disinfection method and improved hand hygiene) could reduce the risk of transmission from fomites and help boost confidence in the use of urban transport [17].

The presence of SARS-CoV-2 in the air is beginning to be investigated and detected even outside the EU [21]. In Italy, according to the Decree of the President of the Council of Ministers on April 26, 2020, good hand hygiene and a high standard of sanitation on public transport were strong recommendations for the use of buses and trains during the pandemic.

Our results show that buses are more contaminated than trains. During the sampling phases on the trains, we noticed that hydroalcoholic solutions were available to the public in all the railway stations, where many signs invited the frequent use of disinfectant. Moreover, while approaching train stops, audio recordings were played repeatedly, reminding passengers to maintain physical distance, to disinfect their hands and to wear face masks correctly, completely covering the nose and mouth. Human behaviors, risk perception and attention to context play an essential role in the adoption of the most common hygiene practices. Protections for workers include the use of barcode and QR code readers for checking tickets, which eliminates the need to touch paper tickets, thus avoiding any contact with passengers. 

Our results disagree with those of Di Carlo et al. [16], whose study found no positive SARS-CoV-2 results. This could be explained by the fact that the authors conducted the study during the lockdown period, when the circulation and use of public transport was extremely limited to particular cases. In addition, the bus company made the wearing of gloves a strict requirement for entering the vehicle.

The most contaminated surfaces were handrails. The high level of positivity found on this type of surface could be linked to passengers holding onto the handrail for stability when entering or exiting the bus or train. The presence of SARS-CoV-2 on multiple points of the same handrail could be explained by the fact that everyone has a different grip and a different height.

Our data showed that the highest percentage of positive samples was obtained between 10:05 a.m. and 11:00 a.m. on buses. This time slot usually coincides with a greater crowding of municipal means of transport by people who do not have fixed working hours or who go out to shop or to meet relatives and friends. The high frequency of public transport may not allow adequate sanitation procedures to be carried out between one shift and another. We observed a very high correlation between the type of transport and crowd: when trains were more crowded, they were more thoroughly sanitized. Furthermore, sampling was performed on weekdays, and Thursday was the day with the greatest number of positive samples, probably related to a cumulative contamination effect due to the use of inadequate sanitation procedures. In order to reduce or contain the spread of the virus, it would be more appropriate to increase the number of daily trips, especially during the most crowded hours of the day, and to carry out a correct and continuous sanitation of public transport. The results of this study underline the importance of the strategies needed to continue to limit the number of people in public indoor spaces in small, enclosed environments, such as buses, and to require people to wear masks. It is important to be aware of these data in consideration of the desires and need to reopen schools, especially because many students use trains and buses. To reduce the potential infection risks associated with the use of public transport, we believe it is necessary to continue to follow these simple rules, but above all to check the careful application of preventive measures.

This study has some limitations. Surface samples were taken mostly at one time during the day, so we have no information on afternoon travelers. The train and bus routes are not matched, because the cities involved in the studies are served by both trains and buses, but the study used more bus routes.

## 5. Conclusions

The less stringent measures currently in place are allowing an increasing number of people to resume normal daily activities, including the use of public transport. Our results highlight a greater level of contamination on buses than on trains, particularly on handrails used for entering or exiting buses. The presence of the virus on many bus surfaces highlights how the sanitation systems on buses and trains are not sufficient to limit the spread of SARS-CoV-2. Therefore, it would be advisable to review the sanitation protocols both in terms of frequency and products used.

## Figures and Tables

**Table 1:** Main characteristics of the surface samples.

Variable	Test Results		Total
	Positive	Negative	No. (%)
Type of Means			
Bus	29	121	150 (50)
Train	3	147	150 (50)
Point of Retrieval			
Handrails	13	111	124 (41.3)
Seat Handles	10	68	78 (26)
Passage Handle	-	8	8 (2.7)
Door Open/Close Buttons	1	25	27 (9)
Stop Button	8	37	45 (15)
Tables and other surfaces	-	18	18 (6)
Day of the week			
Monday	2	98	100 (33.3)
Tuesday	7	93	100 (33.3)
Thursday	23	77	100 (33.3)
Occupied seats			
Less or equal to 25	4	166	170 (56.7)
More than 25	28	102	130 (43.3)
Company			
A	1	19	20 (6.7)
B	14	46	60 (20)
C	12	18	30 (10)
D	2	38	40 (13.3)
E	3	147	150 (50)

**Table 2:** Results of the univariable and multivariable logistic models to evaluate effect of main characteristics of the tested samples.

Variable	Univariable Analysis					Multivariable Analysis		
	Model Fit Information AIC (the Lower the Better)	Wald Chi-Square for the Effect of the Variable	*p*-Value	Classes Compared *	OR (95% CI)	Wald Chi-Square for the Effect of the Variable	*p*-Value	OR (95% CI)
						AIC: 156.8; Chi-Square: 34.54, *p* Value < 0.0001)		
Type of transport	180.7	15.84	<0.0001	Train vs. bus	11.7 (3.5–39.5)	17.55	<0.0001	14.3 (4.1–49.5)
Day of the sample	184.2	18.74	<0.0001	Monday vs. Thursday	0.07 (0.02–0.3)	20.59	<0.0001	0.06 (0.01–0.2)
				Tuesday vs. Thursday	0.25 (0.1–0.6)			0.2 (0.08–0.5)
Class of seats	177.36	19.62	<0.0001	More than 25 seats vs. less or equal to 25 seats	0.08 (0.03–0.26)	Removed from the model by the stepwise procedure		
Routes	164.9	29.22	0.0011	BT-BA vs. BA-FG	32.2 (3.1–333.3)	Removed from the model by the stepwise procedure		
				CA-AND vs. BA-FG	40 (4.6–333.3)			
				CO-AND-BT vs. BA-FG	32.2 (3.7–333.3)			
				BT-BA vs. BA-FG	16.1 (2.4–111.1)			
				CA-AND vs. BA-FG	19.6 (3.7–100)			
				CO-AND-BT vs. BA-FG	16.1 (3–83.3)			
Company	168.8	32.42	<0.0001	A vs. E	2.6 (0.3–26.1)	Removed from the model by the stepwise procedure		
				B vs. E	14.9 (4.1–54.1)			
				C vs. E	32.6 (8.4–126.7)			
				D vs. E	2.6 (0.4–15.9)			
Point of retrieval	207.3	0.19	0.9785	--	--	Not used in the multivariable model		
		0.0007	0.9794	Handrails	--			
		0.0007	0.9786	Handles	--			
		0.0008	0.9781	Buttons	--			
Time of the run	174.1	16.04	0.1892	--	--	Not used in the multivariable model		

*: the OR reported for variables with more than two classes are relative only to statistically significant comparison between pairs. LEGEND: BA = Bari; BT = Barletta; AND = Andria; FG = Foggia; CO = Corato.
